# Growth and characterization of CNT–TiO_2_ heterostructures

**DOI:** 10.3762/bjnano.5.108

**Published:** 2014-07-02

**Authors:** Yucheng Zhang, Ivo Utke, Johann Michler, Gabriele Ilari, Marta D Rossell, Rolf Erni

**Affiliations:** 1 Electron Microscopy Center, EMPA, Swiss Federal Laboratories for Materials Science and Technology, Überlandstrasse 129, CH-8600 Dübendorf, Switzerland; 2 Laboratory of Mechanics of Materials and Nanostructure, EMPA, Swiss Federal Laboratories for Materials Science and Technology, Feuerwerkstrasse 39, CH-3602 Thun, Switzerland

**Keywords:** atomic layer deposition (ALD), carbon nanotubes, electron energy loss spectroscopy (EELS), interface, titanium dioxide (TiO2), transmission electron microscopy (TEM)

## Abstract

A thriving field in nanotechnology is to develop synergetic functions of nanomaterials by taking full advantages of unique properties of each component. In this context, combining TiO_2_ nanocrystals and carbon nanotubes (CNTs) offers enhanced photosensitivity and improved photocatalytic efficiency, which is key to achieving sustainable energy and preventing environmental pollution. Hence, it has aroused a tremendous research interest. This report surveys recent research on the topic of synthesis and characterization of the CNT–TiO_2_ interface. In particular, atomic layer deposition (ALD) offers a good control of the size, crystallinity and morphology of TiO_2_ on CNTs. Analytical transmission electron microscopy (TEM) techniques such as electron energy loss spectroscopy (EELS) in scanning transmission mode provides structural, chemical and electronic information with an unprecedented spatial resolution and increasingly superior energy resolution, and hence is a necessary tool to characterize the CNT–TiO_2_ interface, as well as other technologically relevant CNT–metal/metal oxide material systems.

## Introduction

Since the discovery by Iijima in 1991, carbon nanotubes (CNTs) have always been on the research frontier due to their extraordinary properties [1–5]. Applications based on CNTs including high sensitivity sensors, next generation transistors and high efficiency solar cells have been proposed, and tremendous progress has been achieved. A particularly interesting research area is to combine CNTs with other inorganic nanomaterials, i.e., metal and metal oxide nanoparticles, and to exploit unique properties of each component to realize a synergetic effect of the ensemble. For example, while it has been long discovered that TiO_2_, a semiconductor that can be obtained cost-effectively and environmentally friendly, is a good candidate for electrochemical photocatalysis [6], recent research shows that its limited absorption of only the UV part of the sunlight spectrum can be extended to visible light when forming a nanocomposite with CNTs [7–9]. This can significantly increase the efficiency of electrochemical photocatalysis, and benefits largely in areas such as acquiring sustainable energy and preventing environmental pollution.

In this context, this paper reviews growth of metal and/or metal oxide particles on CNTs and the characterisation by using analytical transmission electron microscopy (TEM) techniques with a focus on the TiO_2_–CNT material system. The mechanisms of efficiency enhancement for photocatalysis by using the ensemble are reviewed. Methods to fabricate nanocomposites consisting of CNTs and metal/metal oxides are surveyed. In particular, an atomic layer deposition (ALD) has recently been used to deposit TiO_2_ nanoparticles on CNTs in a controllable fashion. Characterization of the interface will help to understand the mechanisms, and requires techniques capable of revealing structural details on a nanometer and atomic scale, for which electron energy loss spectroscopy (EELS) is a good candidate. The physical principle of EELS is explained, and a survey of applying EELS to characterization of several technologically important nanomaterials including CNTs and metal/metal oxide nanoparticles is presented. The challenges of studying the TiO_2_–CNT interface using EELS are also discussed.

## Review

### Photocatalysis using TiO_2_–CNT

**Figure 1:**
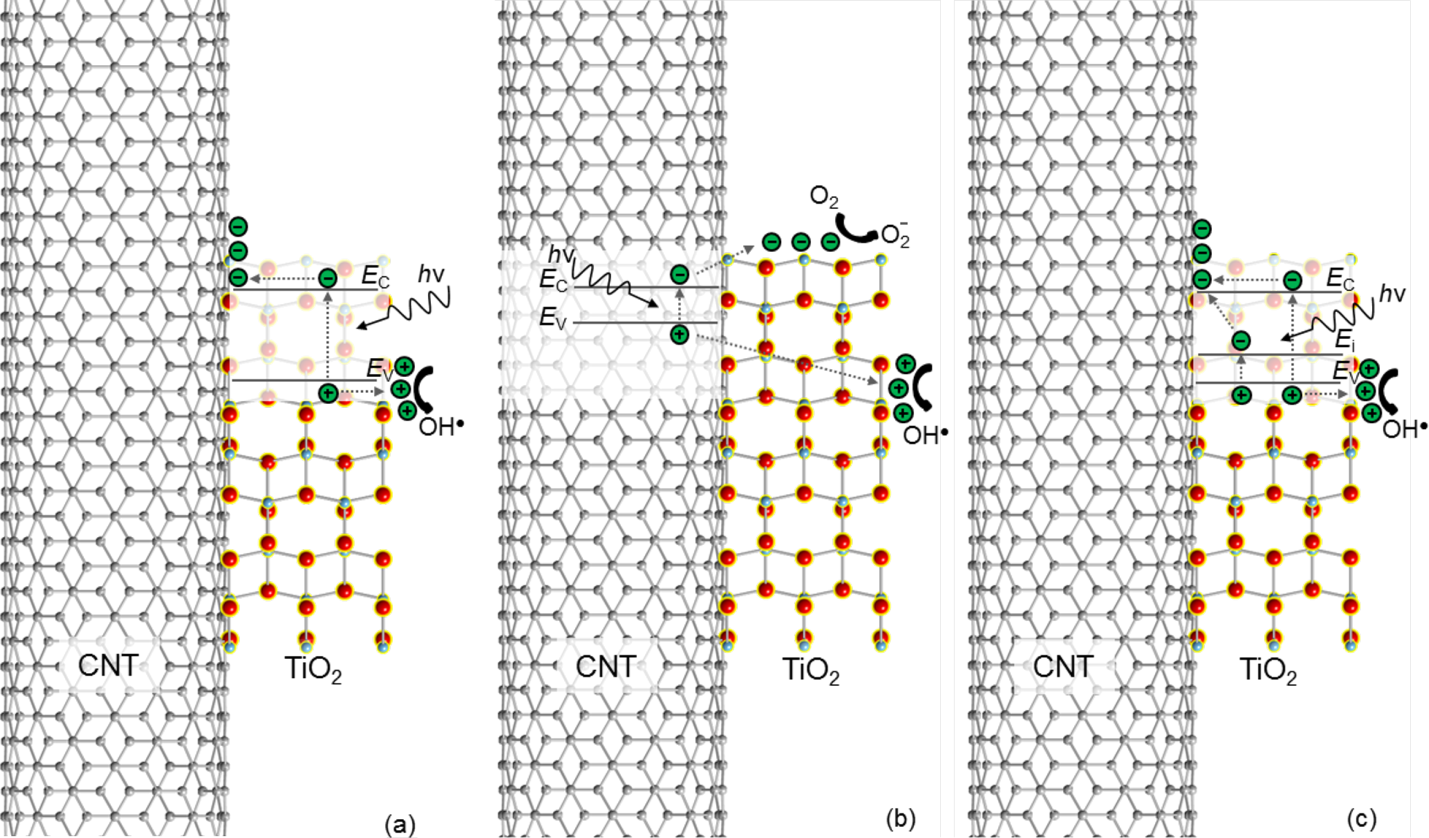
Efficiency enhancement mechanisms for photocatalysis using CNT–TiO_2_ nanocomposites. (a) CNT scavenges electrons generated in TiO_2_, resulting in excessive holes on the surface for redox actions. (b) CNT acts as a photosensitizer and injects electrons and holes into TiO_2_ for redox actions. (c) CNT introduces energy states in the band gap of TiO_2_ which can convert long wavelength photons to electrons and holes. Adapted from [18]. Copyright (2009) WILEY-VCH Verlag GmbH & Co. KGaA, Weinheim.

Among all the nanocomposites consisting of CNTs and metal/metal oxides, CNT–TiO_2_ has attracted most research interest because of its unique properties and potential applications such as photocatalysis, especially since the recent discovery that with the addition of CNTs, the light absorption of TiO_2_ can be extended to the visible-light region [8–14]. This can significantly increase the photocatalytic efficiency. In literature several photocatalysis enhancement mechanisms based on TiO_2_–CNT have been proposed, as illustrated in Figure 1. Hoffmann et al. have proposed that electrons and holes are generated in TiO_2_ after absorption of photons, and CNTs act as an electron scavenger due to their high electron storage capacity and conductivity, so that excessive holes are generated on the surface of TiO_2_ for redox reactions [12]. Alternatively, Wang et al. propose that electrons and holes are generated in the semiconducting CNTs, which act as a sensitizer [9]. The electrons are injected into the conduction band of TiO_2_ to form superoxide radicals and the holes into the valence band of TiO_2_ to form hydroxyl radicals. The third mechanism was proposed by Pyrgiotakis [13], in which the C–O–Ti bond introduces energy states in the band gap of TiO_2_, and is attributed to the extended absorption of longer wavelength light. In addition, they also found that the electronic structure of CNTs may have a bigger effect on the photocatalytic performance than the chemical bond between CNTs and TiO_2_, since the arc-discharge-synthesized CNTs show a dramatically higher photocatalytic dye degradation rate than the CVD-synthesized CNTs, which is attributed to the smaller number of defects in the multi-wall (MW) tubes of the former. In reality, there are probably more than one mechanism acting in the photocatalysis. It is, however, crucial to investigate the interface so as to understand the dominant mechanism. A fundamental understanding of the mechanisms can also benefit other applications such as photoelectrochemical cells [15], sensors [16] and batteries [17] where the synergetic effect of the ensemble can be fully exploited.

It is therefore of paramount importance to achieve CNT–TiO_2_ in a controllable way and further characterize the interface. Fundamental questions need to be answered, such as how TiO_2_ nucleates and grows on CNTs, and what bonding is formed at the interface. Growth methods with a nanoscale precision are required to fabricate the ensemble. Characterization methods that can provide structural, chemical and electronic information on a nanometer or atomic scale are sought to investigate the interface. Some of the recent progress achieved in these areas is reviewed here.

### ALD growth of CNT–TiO_2_

**Figure 2:**
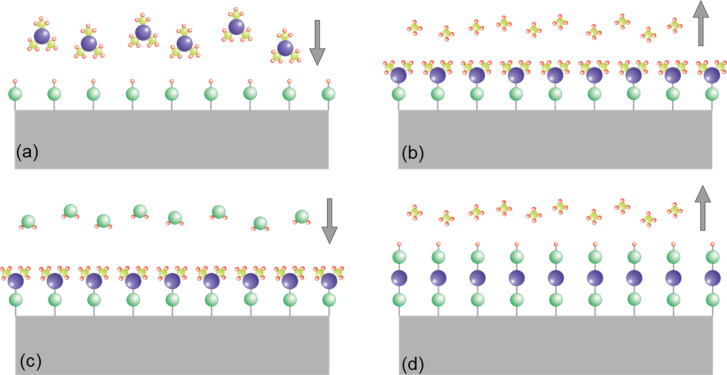
A schematic illustration of one cycle in ALD: (a) The first precursor flows into the chamber and reacts on the active sites of the substrate by partially losing ligands. (b) One mono-layer of reactant is formed and the chamber is purged to remove the remaining precursor and the intermediate product. (c) The second precursor is then introduced into the chamber to react with the remaining ligands and to form the desired compound molecule. (d) One cycle is complete when all of the ligands of the first precursor are consumed and one mono-layer of the compound is formed. The chamber is purged again for next cycle.

Many methods have been developed to fabricate CNT–metal/metal oxide nanocomposites. For CNT–TiO_2_, the methods that are mostly adopted are sol–gel and hydrothermal deposition. A good summary of the synthesis methods can be found in the review by Leary [19]. Recently, we have adopted the atomic layer deposition (ALD) technique to deposit TiO_2_ on CVD-grown MW-CNTs. ALD relies on self-limiting surface reactions (dissociative chemisorption) of gases which are alternately introduced into and purged out of the reaction chamber. The surface reaction will establish a chemical bond of the precursor molecule with the substrate through the reaction of an organic ligand to a volatile compound. The remaining organic ligands still bound to the metal atom prevent further chemisorption. This self-limitation has two important consequences which make ALD very attractive: a) The thickness of deposited material will be limited to a monolayer at maximum, i.e., there is atomistic control of the thickness, and b) ultra-high aspect ratio structures can be deposited conformally as one can give enough time for the molecules to diffuse into the porous volume without clogging the surface. In order to continue the process the organometallic precursor gas needs to be purged and a second gas, the reactant will be introduced and reacting in a self-limiting manner with the remaining organic ligands. As a result of this full ALD cycle, the ligands react to volatile compounds and the reacted bonds constitute the reaction sites for the next ALD cycle starting again with the organometallic precursor. A schematic illustration of ALD is shown in Figure 2. For titanium isopropoxide Ti(OCH(CH_3_)_2_)_4_ (TTIP) and H_2_O the self-limiting surface reactions in Equation 1 and Equation 2 were proposed. TTIP/H_2_O ALD results in amorphous TiO_2_ for substrate temperatures between 100 and 150 °C while for substrate temperatures higher than 180 °C the crystalline anatase phase is obtained.

[1]
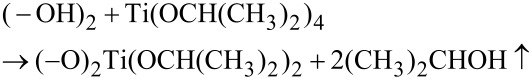


[2]
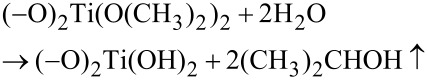


Generally, the organo-metallic precursor molecules for metal oxide ALD, e.g., trimethylaluminium for Al_2_O_3_, titanium tetraisopropoxide for TiO_2_, diethylzinc for ZnO, or tetrakis(ethylmethylamido)hafnium for HfO_2_, need surfaces that are terminated with a functional group, which would allow for their dissociative chemisorption reaction. Hydroxy-(OH)-group terminated surfaces prevailing on many metaloxide surfaces are ideally suited [20–21]. However, single-wall (SW) carbon nanotubes, graphene, and a few noble metals do not possess naturally such a functional-group termination but rather inert surfaces and ALD nucleation is prohibited [22]. Hydrogen-terminated surfaces are also non-reactive and are used to render the ALD process selective in self-assembled monolayer molecules [23]. Only surface defects on CNTs and graphene allow for the chemisorption of metal-organic precursor molecules.

Basically, surface functional groups need to be generated on pristine single-wall CNTs or graphene by specific treatments which attack the surface and introduce defects [24]. The treatments can be divided into covalent and non-covalent functionalization. An ozone treatment, as an example for covalent functionalization, is simple to integrate into the ALD process and was shown to result in conformal Al_2_O_3_ coatings on graphene when using trimethylaluminium and ozone half-cycles, while the use of water resulted in inhomogeneous films [25]. This is due to the generation of oxygenated functional groups on the CNT surface. Also treatments of CNTs prior to ALD by using acids such as H_3_PO_4_ (together with a chromic acid) and HNO_3_ triggered conformal growth of ruthenium [26] and SiO_2_, TiO_2_, and Al_2_O_3_ (after thermal annealing) [27]. Such surface defects seem to be naturally present on multi-wall CNTs with a given surface density. They are responsible for local metal oxide nucleation. The density of oxygenated defects can be increased by oxygen plasma treatment. A conformal film is obtained when the nanocrystals coalesce due to lateral growth after approximately 50–100 cycles (depending on the defect density) [28]. Similar results were obtained with Al_2_O_3_ coatings by using trimethylaluminium and water ALD half-cycles [29] and tetrakis(ethylmethylamido)hafnium with water [30]. In contrast, thin conformal metal oxide ALD films can be easily obtained on organic polymers like polystyrene. Conformal 20 nm thin ZnO coatings were deposited using diethylzinc half-cycles alternated with water on large area polystyrene bead arrays [31]. After removing the polystyrene, a transparent, electrically conductive, hollow sphere array was obtained on top of which an urchin-inspired nanobuilding block design of a solar cell with n-type ZnO nanowires could be realized by using electrochemical deposition [32].

Non-covalent surface functionalization leaves the pristine CNTs sp^2^ structure and carbon atom conjugation intact. Examples include in-situ NO_2_ physisorption which permitted the uniform growth of Al_2_O_3_ on SW-CNT [33], MW-CNT [34] and graphene [35], as well as the physisorption of ethanol and sodium dodecylsulfate on CNTs, which also permitted the ALD of Al_2_O_3_ [36]. So far only amorphous TiO_2_ was deposited by ALD on graphene and CNTs by using an ALD interlayer of Al_2_O_3_ [37].

**Figure 3:**
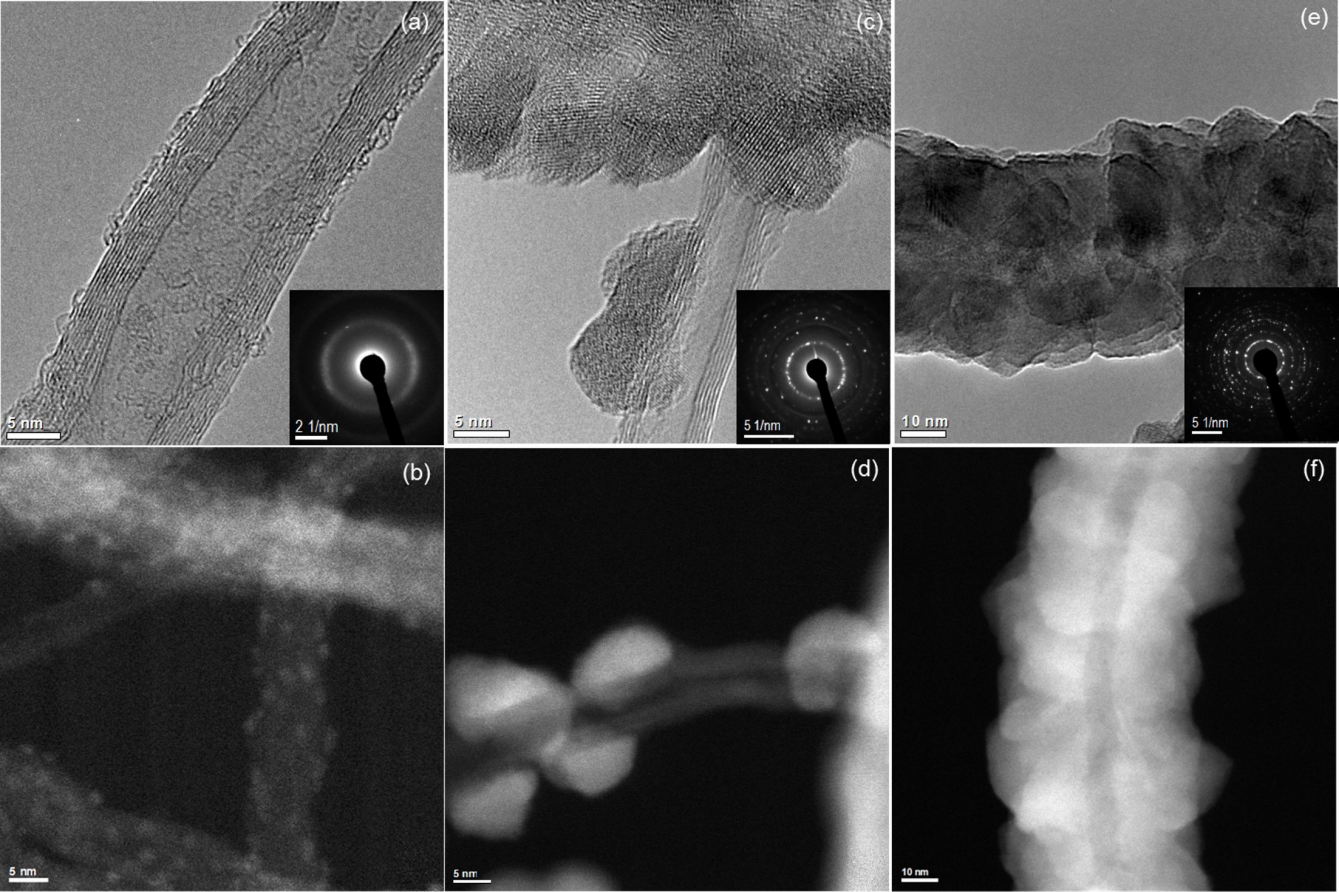
Micrographs of TiO_2_ deposited on MW-CNT by using ALD with different number of cycles to control the particle size. (a), (c) and (e) are the high-resolution TEM images after 20, 200 and 750 cycles, respectively. The inset shows selected area diffraction pattern (SADP). (b), (d) and (f) are the respective STEM-HAADF images providing a higher contrast between the TiO_2_ particles and the MW-CNTs.

By using this method, TiO_2_ of various crystallinity, size and morphology on MW-CNTs has been recently obtained in a controllable way. An example is shown in Figure 3, in which the deposition of TiO_2_ for various numbers of cycles at 220 °C is presented. The morphology changes from small nuclei with diameters smaller than 2 nm after 20 cycles, to clustering of crystalline particles of about 5 nm after 200 cycles, until the formation of a coalesced and conformal layer of an anatase phase after 750 cycles. This provides an ideal scenario to study nucleation and growth of TiO_2_ on CNTs, and especially assisted with the powerful TEM techniques, to investigate the interface that is crucial to applications based on the ensemble. In addition to the growth parameters in ALD, such as temperature and number of cycles, pre-treatment of the surface of the CNTs by using O_2_ plasma is found to have an effect on the nucleation and morphology of TiO_2_. The nuclei density is significantly increased after the surface treatment, probably due to the fact that the plasma introduces more defect sites on the tube-walls, which facilitate the nucleation. A similar effect has been previously reported when depositing various metals such as Ni, Cu, and Pt on MW-CNTs pre-treated with O_2_ plasma [38–39].

### Analytical-TEM characterization of functional nanomaterials

**Figure 4:**
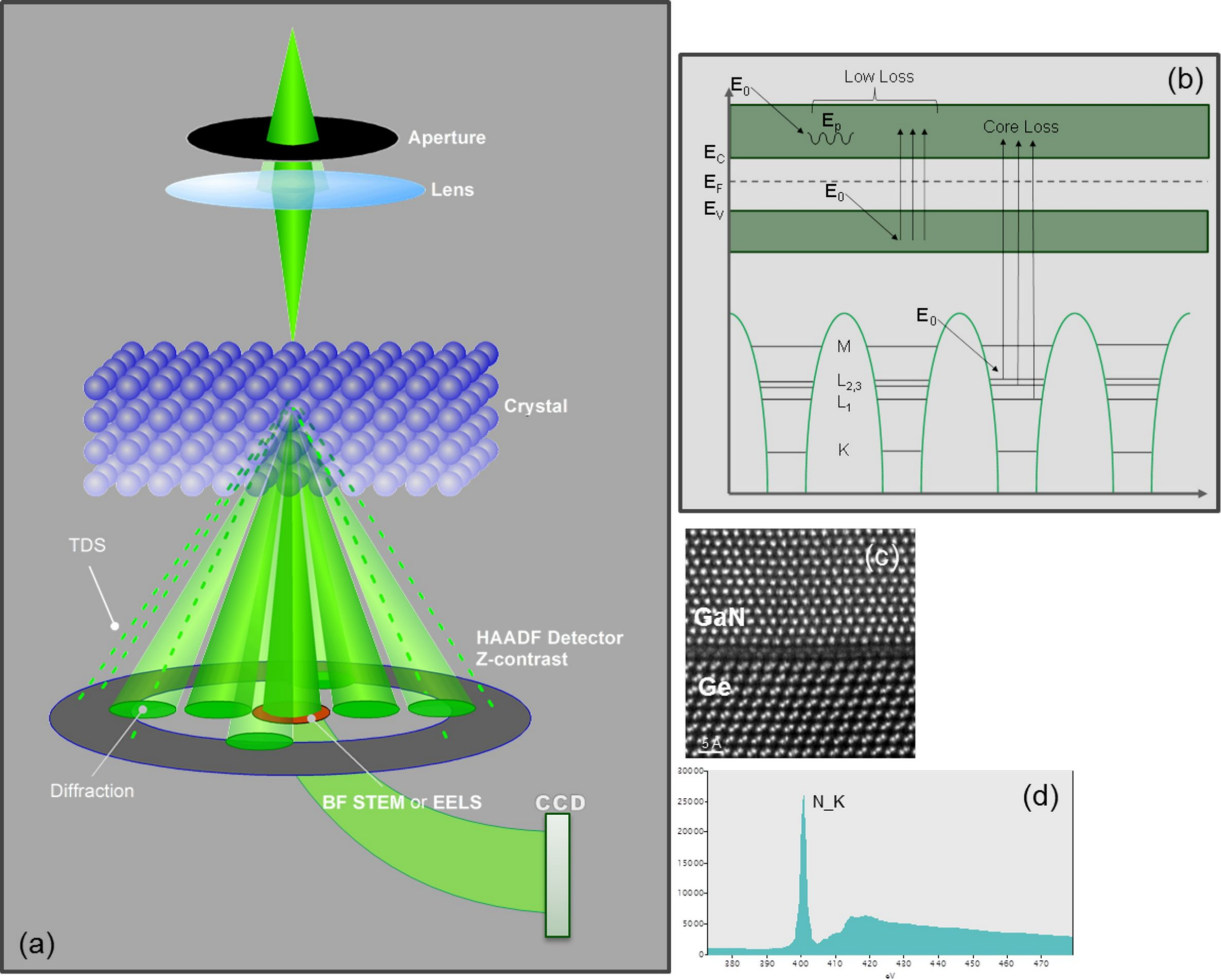
A schematic illustration of the principle in STEM-EELS: (a) the microscope configuration in the STEM mode; (b) the inelastic scattering processes in the TEM sample contributing to the low-loss and the core-loss EELS signals; (c) an atomic-resolution STEM-HAADF image of a GaN–Ge interface; (d) a EELS core-loss spectrum showing the N_K edge of gaseous N_2_.

To fundamentally understand the interface between CNTs and metal/metal oxides on the nanometer or even on the atomic level, TEM plays an irreplaceable role due to its high spatial resolution. With the development of spherical aberration correctors, atomic-resolution imaging at a sub-0.5 Å level has already been demonstrated [40]. On the other hand, analytical TEM provides chemical and electronic information about nanomaterials with an increasingly improved energy resolution. One powerful analytical TEM technique is electron energy loss spectroscopy (EELS). It detects electrons that lose a certain amount of energy due to inelastic scattering processes inside the specimen. Depending on the physical nature of the inelastic scattering processes, the EELS spectrum can be divided into two regions: the low-loss region, in which the energy loss, ∆*E*, is typically less than 50 eV, and the core-loss region, in which ∆*E* is above 50 eV. The physical principles of the inelastic scattering processes in EELS are schematically illustrated in Figure 4.

The low-loss region reflects a collective excitation of electrons in the conduction band, known as plasmons, as well as single electron excitations in the outer shells, i.e., excitations from the valence band to the conduction band. Hence, the low-loss signal contains information about the band structure of the specimen, and has been used to determine the band gaps of semiconductors [41]. Based on a dielectric model, the dielectric function can be extracted from the low-loss region by using a Kramers–Kronig analysis and its energy dependence can be measured at wavelengths beyond optical methods. However, the low-loss signals are complicated by other inelastic processes such as surface plasmons and retardation loss due to the Čerenkov emission, such that simulations based on a first-principle calculation is difficult [42]. Additionally, the spatial resolution of the low-loss is limited by the delocalization of the scattering processes, and it is yet to measure the low-loss signals on an atomic level.

**Figure 5:**
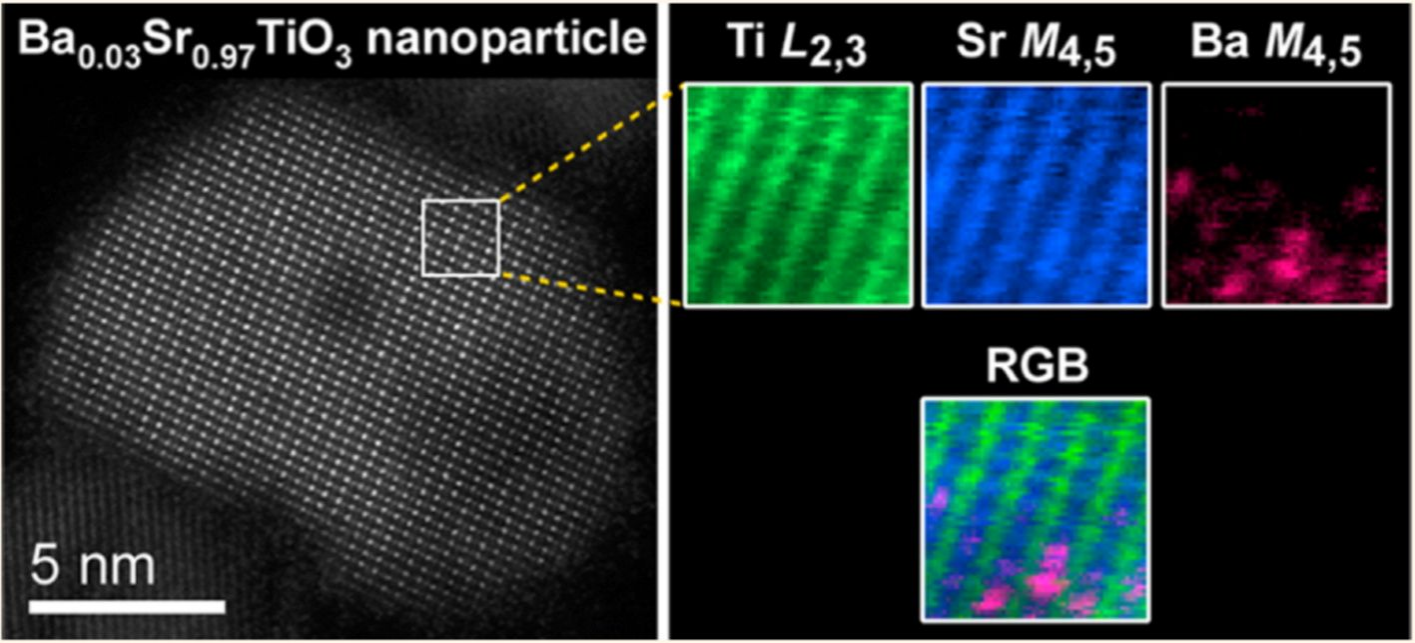
An atomic-resolution chemical mapping of Ba-doped SrTiO_3_ nanoparticles: (a) the HAADF-STEM image of a Ba-doped STO nanoparticle along the [011] direction; (b) the simultaneous chemical mappings measuring the Ti_L_2,3_, Sr_M_4,5_, and Ba_M_4,5_ edges, respectively, and the corresponding RGB map. Reprinted with permission from [49]. Copyright (2012) The American Chemical Society.

The core-loss region contains the electrons that lose energy by exciting the core-shell electrons in the specimen. Because the energy levels in the core-shells are unique for every element, the core-loss signals can be used as a fingerprint of the elements present in the specimen, with a much higher energy resolution than energy dispersive X-ray (EDX) analysis. The core-shell excitation process is localized around the atoms, and hence atomic-resolution chemical mappings can be achieved by using EELS core-loss signals combined with a sub-nanometer sized electron probe [43–47]. For example, by using EELS at a low acceleration voltage of 60 kV to avoid beam damage, Suenaga et al. have been able to detect single calcium atoms inside the metallofullerence-doped single-wall nanotubes [46]. Nakagawa et al. have applied the atomic-scale EELS to the study of the (001) LaAlO_3_ and SrTiO_3_ interfaces, and observed an asymmetry between ionically and electronically compensated interfaces, which explains why some semiconductor interfaces cannot be atomically sharp [48]. Recently, Rossell et al. [49] have adopted STEM-EELS combined with multivariate statistical analysis (MSA) to map the distribution of Ba dopant atoms in SrTiO_3_ nanoparticles, as shown in Figure 5. The results provide direct evidence for clustering of the Ba atoms in the nanoparticles, which may explain the presence of the polar nanoregions in Ba:SrTiO_3_. These results demonstrate the capability of EELS to characterize the interface of CNT–metal/metal oxides on the nanometer or the atomic level.

The electron loss near-edge fine structures (ELNES) in EELS provide additional information about the chemical and electronic environment of the ionized atoms. The spectrum region corresponds to the electrons with an energy loss within about 50 eV above the edge threshold. An example of ELNES at the N_K edge of N_2_ is shown in Figure 4d. Within a certain approximation, the observed details represent a symmetry-projected density of unoccupied states above the Fermi level [50]. Hence, ELNES is very sensitive to the local atomic coordination and can be used to probe atomic bonding. The same element can have different ELNES signals if it is in different chemical states. Performed in the STEM mode, ELNES can provide the electronic information on a nanometer or atomic scale. It is an ideal technique to study CNTs and related nanomaterials. For example, by measuring the ratio of π* and σ* peaks at the C_K edge, the amount of sp^2^ and sp^3^ hybridizations can be quantified in amorphous carbon, which determines the physical properties [51–52]. Similarly, Muller et al. have used the ELNES signals to produce a map of sp^2^ and sp^3^ at the interface of diamond grown on Si/SiO_2_ with a sub-nanometer spatial resolution, which helps to understand the nucleation of diamond [53]. Suenaga et al. have performed in-situ bending of SW-CNTs in TEM and observed a change in the C_K edge ELNES at kinks of the CNT bundles, indicating the change of the electronic structure with the deformation [54]. Theoretical calculations based on density functional theory (DFT) can be used to simulate the details in ELNES and to fundamentally predict the atomic and the electronic structure. Depending on the atomic potentials defined in the calculation, methods based on the band theory, the molecular orbitals or the multiple scattering are developed, either in the reciprocal space or in the real space, to simulate the ELNES of both crystalline and amorphous materials [50]. Titantah et al. performed DFT calculations of ELNES on CNTs, taking into consideration the effect of curvatures, the electron-beam orientation as well as the inclusion of the core hole [55]. Their calculation also predicted an energy shift of 5 eV in the σ* peak if the C–C bond length is increased to 1.7 Å, demonstrating the sensitivity of ELNES to the bond length [56]. Using EELS at a high energy resolution, Brydson et al. observed a difference in the ELNES of the Ti_L_2,3_ and O_K edges between anatase and rutile TiO_2_, which could be modelled using real-space multiple-scattering calculations [57].

With all the advancements in the analytical TEM techniques and their application to characterization of carbon-based nanomaterials and metal/metal oxides, it is now possible to study the interface of the nanocomposites formed of the two material systems on a nanometer or even atomic level, which is a determining factor for the nanodevices based on the ensemble. Recently, Ilari et al. have studied the interface between graphite and metals including Ni and Cu by using STEM-EELS, as well as between MW-CNTs and the metals, by using STEM-EELS with an electron probe down to sub-nanometer [58]. The interaction at the interface was found to be different for Ni and Cu, leading to different interfacial structures. The study helps to understand the behavior of metallic contacts on CNTs, a key step for electronic devices at a nanometer scale. Similarly, analytical TEM can also be used to study the interface between TiO_2_ and CNTs. In particular, the Ti_L_2,3_ and C_K edges in EELS contain important atomic and electronic information about the interface, which can shed light on the performance enhancement mechanisms in photocatalysis that is explained in the proceeding section. Particularly, with a high spatial resolution STEM-EELS can be used to probe the bonding at the interface between CNTs and the nanoparticle and, answer the question if any covalent bonds such as C–O or C–Ti are formed at the interface. Other analytical techniques such as X-ray photoelectron spectroscopy (XPS) [59] and Raman spectroscopy [60] can also provide information about the interfacial bonding, however on a much larger scale. The bonding information obtained using STEM-EELS on a nanometer or even on an atomic scale, when combined with first-principle calculation, can help to learn the atomic and electronic structure at the interface, which is crucial to understanding charge transfer and hence the working mechanisms of the applications including photocatalysis and photovoltaics based on the ensemble.

**Figure 6:**
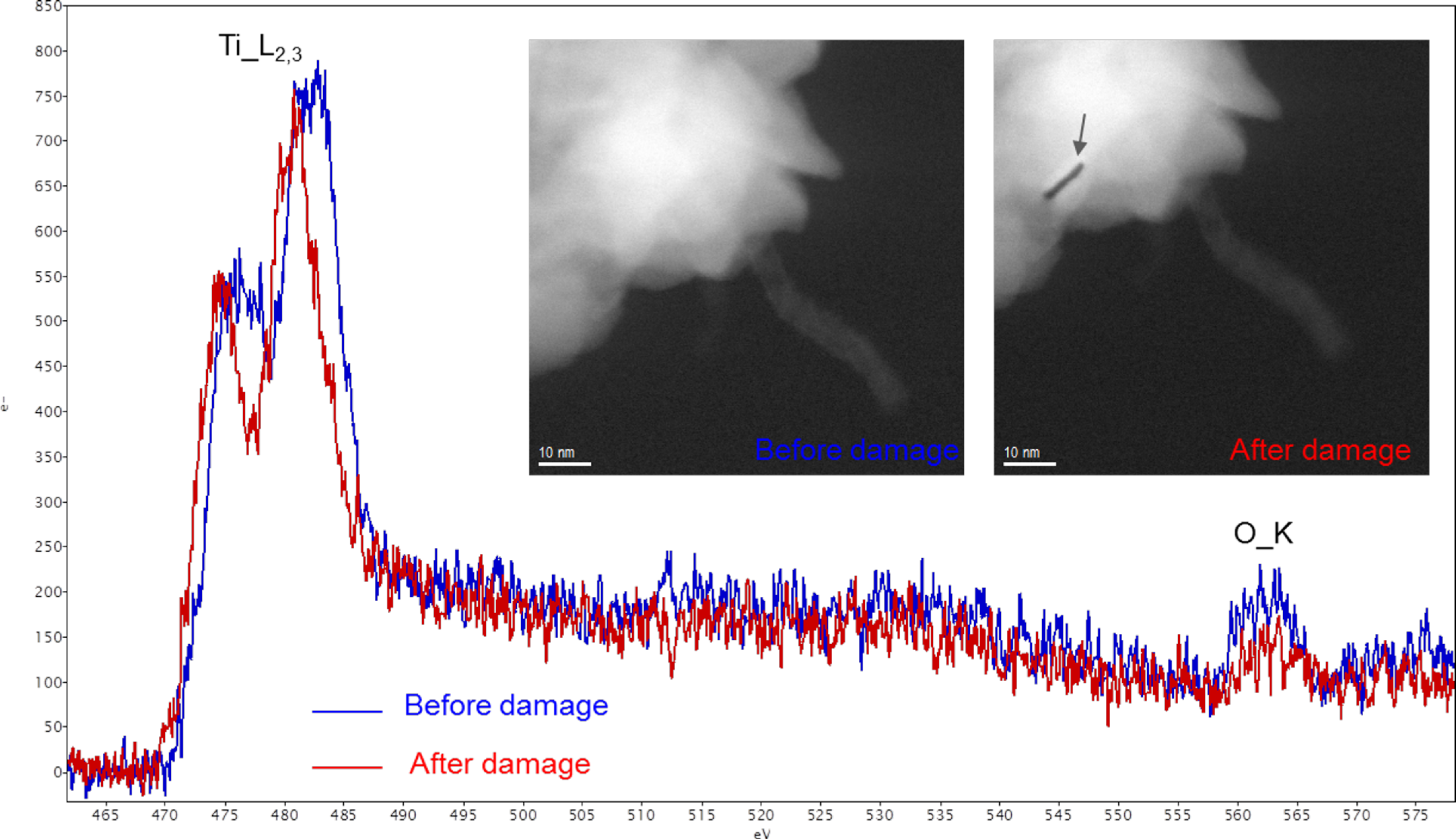
An example of electron beam damage during EELS acquisition. Note that there is a chemical shift in the Ti_L_2,3_ edge after the damage. The damage is also visible in the STEM-HAADF image.

While analytical TEM, in particular EELS, is a powerful tool to study the interface between CNTs and metal/metal oxides, the task is quite challenging and several difficulties need to be overcome. The damage in the sample caused by the high energy electron can hinder acquisition of sufficient EELS signals. For example, as shown in Figure 6, it was found that the ALD-grown TiO_2_ on MW-CNTs can be damaged only a few seconds after the beam is incident on the sample. The Ti_L_2,3_ edge from the damaged TiO_2_ shows a red-shift due to sputtering of oxygen atoms by the high energy electrons and the resulting change of the oxidation state [61]. The damage mechanism can however depend on the specific chemical environment in the sample. To avoid the damage, optimized experimental conditions are required, such as a lower acceleration voltage or a lower probe current density. This should be balanced with a reasonable acquisition time to minimize the carbon contamination that can also be an obstacle especially when studying carbon-based nanomaterials. In addition, it is more difficult to study interfaces consisting of CNTs than those in semiconductor thin films due to the curvature of the former. Especially for CNTs of small diameters, the signals at the interface may be embedded in those from the volume, due to 2D projection of TEM characterization While the length of CNTs does not affect the measurement, the radius can be an important factor, since the smaller the radius, the larger the curvature. The difficulty can be overcome as the spatial resolution and the chemical sensitivity of EELS is continuously increasing. Additionally, combined with electron tomography, it may be possible to use EELS to learn about the interfacial structure in 3D on a nanometer scale [62–63].

## Conclusion

Nanocomposites combining CNTs and metal/metal oxides can achieve synergetic functions beyond the capability of each component. However, successful implementation relies on growing the ensemble with a good control and understanding the interface through advanced characterization. For the material system CNT–TiO_2_, ALD offers the good control of growth conditions to obtain different morphology, size and crystallinity of TiO_2_ on CNTs, which not only facilitates the study of nucleation mechanisms, but also is beneficial to various applications. As an important characterization tool for nanomaterials, EELS can be used to study the interface and gain fundamental knowledge of how CNTs interact with metal/metal oxides on a nanometer or atomic level. Difficulties exist when applying the growth and characterization methods, but can be overcome with the rapid advancement of both the techniques and the instrumentations.
